# New Insights into the Runt Domain of RUNX2 in Melanoma Cell Proliferation and Migration

**DOI:** 10.3390/cells7110220

**Published:** 2018-11-20

**Authors:** Michela Deiana, Luca Dalle Carbonare, Michela Serena, Samuele Cheri, Francesca Parolini, Alberto Gandini, Giulia Marchetto, Giulio Innamorati, Marcello Manfredi, Emilio Marengo, Jessica Brandi, Daniela Cecconi, Antonio Mori, Maria Mihaela Mina, Franco Antoniazzi, Monica Mottes, Natascia Tiso, Giovanni Malerba, Donato Zipeto, Maria Teresa Valenti

**Affiliations:** 1 Department of Neurosciences, Biomedicine and Movement Sciences, University of Verona, I-37134 Verona, Italy; michela.deiana@univr.it (M.D.); michela.serena@univr.it (M.S.); samuele.cheri@univr.it (S.C.); francesca.parolini@univr.it (F.P.); antonio.mori@univr.it (A.M.); monica.mottes@univr.it (M.M.); giovanni.malerba@univr.it (G.M.); donato.zipeto@univr.it (D.Z.); 2 Department of Medicine, University of Verona, I-37134 Verona, Italy; luca.dallecarbonare@univr.it (L.D.C.); giulia.marchetto@univr.it (G.M.); mariamihaela.mina@univr.it (M.M.M.); 3 Department of Surgery, Dentistry, Pediatrics and Gynecology, University of Verona, I-37134 Verona, Italy; alberto.gandini@univr.it (A.G.); giulio.innamorati@univr.it (G.I.); franco.antoniazzi@univr.it (F.A.); 4 Department of Sciences and Technological Innovation, University of Piemonte Orientale, I-15121 Alessandria, Italy; manfredi@uniupo.it (M.M.); emilio.marengo@uniupo.it (E.M.); 5 Proteomics and Mass Spectrometry Laboratory, Department of Biotechnology, University of Verona, I-37134 Verona, Italy; jessica.brandi@univr.it (J.B.); daniela.cecconi@univr.it (D.C.); 6 Department of Biology, University of Padova, I-35131 Padova, Italy; natascia.tiso@unipd.it

**Keywords:** RUNT, RUNX2, CRISPR/Cas9, melanoma

## Abstract

The mortality rate for malignant melanoma (MM) is very high, since it is highly invasive and resistant to chemotherapeutic treatments. The modulation of some transcription factors affects cellular processes in MM. In particular, a higher expression of the osteogenic master gene RUNX2 has been reported in melanoma cells, compared to normal melanocytes. By analyzing public databases for recurrent RUNX2 genetic and epigenetic modifications in melanoma, we found that the most common RUNX2 genetic alteration that exists in transcription upregulation is, followed by genomic amplification, nucleotide substitution and multiple changes. Additionally, altered RUNX2 is involved in unchecked pathways promoting tumor progression, Epithelial Mesenchymal Transition (EMT), and metastasis. In order to investigate further the role of RUNX2 in melanoma development and to identify a therapeutic target, we applied the CRISPR/Cas9 technique to explore the role of the RUNT domain of RUNX2 in a melanoma cell line. RUNT-deleted cells showed reduced proliferation, increased apoptosis, and reduced EMT features, suggesting the involvement of the RUNT domain in different pathways. In addition, del-RUNT cells showed a downregulation of genes involved in migration ability. In an in vivo zebrafish model, we observed that wild-type melanoma cells migrated in 81% of transplanted fishes, while del-RUNT cells migrated in 58%. All these findings strongly suggest the involvement of the RUNT domain in melanoma metastasis and cell migration and indicate RUNX2 as a prospective target in MM therapy.

## 1. Introduction

In the last decade, the incidence of MM has increased considerably as a result of lifestyle and environmental changes. Early detection of skin lesions and surgical treatment before cell dissemination represents a successful therapeutic approach. However, after metastases spreading, effective interventions against melanoma are limited. Despite initial positive responses, cells subsequently become resistant to apoptosis and chemotherapy [1]. Many studies, performed in animal models and in primary tumors, have shed light on the complex background involved in MM metastatic progression [2].

In melanoma, melanocytes escape the control of keratinocytes and produce proteases. Then, the detachment process starts after protease digestion of the extracellular matrix. Melanoma cells invade skin tissue and enter blood vessels, forming metastases at distant sites [2]. In addition, we have found that STMN1, a cytosolic phosphoprotein [3], and SSBP1, involved in p53 transcriptional activity [4], are up- and down-regulated, respectively, in melanoma cells and that their modulation is involved in melanoma transformation [5]. It has also been reported that the mutation rate in melanoma is higher than in other solid malignancies. The most frequently mutated genes are BRAF (leading to the activation of the MAPK pathway that contributes to unlimited cell proliferation), KIT (which encodes for a tyrosine kinase receptor involved in melanocyte’s cell cycle regulation), nRAS (the mutated gene that leads to abnormal signaling transduction in the MAPK pathway), and PTEN (a tumor suppressor gene) [6,7]. Other genes involved in melanoma progression are TERT [8], p53 [9], and the transcription factor MITF (Microphthalmia transcription factor) [10]. Due to its critical role in melanocyte proliferation and differentiation, MITF is considered an important melanoma oncogene. Transcription factors, by acting on gene expression, can affect cellular processes and trigger cellular transformation. Some studies have reported also the involvement of the RUNX2 transcription factor in melanoma.

RUNX2 belongs to the RUNX family, and it is involved in many pathways such as apoptosis, Epithelial Mesenchymal Transition (EMT), and stem cell function, affecting WNT, NOTCH, BMP, and RAS signaling as well [11]. In addition, RUNX2 is involved in angiogenic, metastatic, and osteolytic processes [11]. It has been demonstrated that RUNX2 is able to control the metabolic pathways involved in breast cancer progression by repressing SIRT6 tumor suppressor [12]. Ozaki and coworkers showed that RUNX2 associates with Histone deacetylase 6 (HDAC6), thus preventing the pro-apoptotic activity of p53 through its deacetylation [13]. In particular, the regulation of *KIT* gene by RUNX2 and increased RUNX2 gene expression have been documented in melanoma cells [14,15]. *RUNX2* is the master gene of osteogenic differentiation; it binds DNA as a monomer or, with a higher affinity, as a subunit of the heterodimeric complex formed with CBFβ. It is expressed during the commitment of MSCs to osteogenic differentiation and also in the pre-osteoblast and early osteoblast [16]. *RUNX2* gene is located on chromosome 6; the coding sequence is organized in 8 exons, and its expression is controlled by two promoters. The protein isoforms result from the use of alternative promoters as well as from alternative splicing [16]. However, the DNA-binding RUNT domain, which is highly conserved, remains unchanged [16]. Besides being necessary for osteogenic differentiation, RUNX2 also plays a role in several tumor tissues, including pancreatic cancer, breast cancer, ovarian epithelial cancer, prostate cancer, lung cancer, and osteosarcoma [17]. In thyroid cancer patients, we found that RUNX2 mRNA levels were higher in tumor tissue than in normal tissue [18].

In melanoma, it has been shown that RUNX2 is involved in the regulation of the EMT process [19]. Recently, we found a lower migration ability as well as a downregulation of melanoma cells treated with BEL beta-trefoil lectin [14]. However, some molecular aspects underlying the pathways regulated by the RUNT domain are still unknown in melanoma.

Therefore, with the aim of analyzing the role of the RUNT domain and exploring new oncotargets in melanoma, we deleted this DNA-binding domain by using the CRISPR/Cas9 technique in a melanoma cell line. In particular, we investigated the role of RUNT domain deletion in crucial features such as cell viability as well as migration ability and epithelial mesenchymal transition. In addition, we analyzed the expression of *STMN1* and *SSBP1*. The deletion of the RUNT domain indeed affected the expression of these two genes, which we had shown previously to be involved in melanoma transformation. Our findings further highlight the role of RUNX2 in this malignancy.

## 2. Materials and Methods

### 2.1. Bioinformatic Investigation of RUNX2 and Associated Genes in Melanoma

The cBio genomic Cancer Portal (http://www.cbioportal.org) was used to query [20] the Cancer Genome Atlas database (TCGA, https://cancergenome.nih.gov/) to collect information about gene expression, regulation, and mutations of *RUNX2* in 470 Skin Cutaneous Melanoma (SKCM) patients. This analysis allows one to detect specific biological events, to generate biological pathways involving genes of interest, and to retrieve epidemiological information.

The gene products identified by the cBioportal Network analysis were also submitted to the STRING portal (https://string-db.org/) for independent inspection of their predicted connections.

### 2.2. Cell Cultures

A375 melanoma cells (purchased from American Type Culture Collection—Rockville, MD, USA) were cultured under a humidified atmosphere of 5% CO_2_ and passaged in growth medium: DMEM/F12 containing 10% FBS (fetal bovine serum) supplemented with antibiotics (1% penicillin and streptomycin) and 1% glutamine. Cells were routinely tested for the absence of mycoplasma contamination.

### 2.3. CRISPR/Cas9-Mediated Deletion of the RUNT Domain from RUNX2

CRISPR/Cas9 was used to generate a mutant cell line in which the RUNT domain was deleted from RUNX2. Two specific gRNAs, flanking the deletion, were designed by analyzing the target sequence with both CHOPCHOP [21,22] and MIT (http://crispr.mit.edu/) CRISPR design tools. Two gRNAs with higher efficiency and lower gene off-targets were chosen (gRNA A CCCATCTGGTACCTCTCCGA; gRNA B GATCGTTGAACCTTGCTACT).

The two selected gRNAs were individually cloned in the PX459 V2.0 Cas9 expressing vector (Addgene), following the protocol described by Ran et al. [23]. A375 cells were co-transfected with 1 μg of each plasmid using the Amaxa Nucleofector kit V, following the manufacturer’s protocol. Transfected cells were selected in the presence of 0.2 μg/mL puromycin (Thermo Fisher Scientific, Waltham, MA, USA) for three days. To isolate the edited cells, a single cell cloning was performed. The RUNX2 deletion protein was tested by Western blot. To confirm the deletion in the RUNT domain, the specific RUNX2 genomic region NM_001024630.3, c.424_580, encoding for the DNA binding RUNT domain, was amplified by PCR (FW TGAAGTGGCATCACAACCCA; RV AGTCAGAGACCTACCTCGTC) and the products were purified with the FastGene^®^ extraction kit (Nippon Genetics, Tokyo, Japan). The forward PCR primer was then used for Sanger sequencing using the GenomeLab™ DTCS quick start kit and CEQ 8000 Genetic Analysis System (SCIEX, San Francisco, CA, USA) following manufacturer’s instructions.

### 2.4. XTT Test

Cell viability was evaluated by the reduction of the tetrazolium salt XTT (sodium 3I-[1-phenylamino-carbonyl-3,4-tetrazolium]-bis(4-methoxy-6-nitro) benzene sulfonic acid hydrate- Cell proliferation kit II—XTT Roche) as previously reported [24]. Six replicates in three independent experiments were performed.

### 2.5. Cell Proliferation Test

Proliferating cells nuclei were identified by Ki67-positive immunofluorescence on cells cultured in slide glass chambers, as previously reported [11]. In particular, four different fields were measured for each sample, in three independent experiments with about 80–100 total cells.

### 2.6. TUNEL Test

Apoptosis was analyzed in a glass chamber with the TUNEL technique using terminal deoxynucleotidyl transferase (TdT) (ApoTag Fluorescein In Situ Apoptosis Detection Kit, S7110, Millipore Corporation, Billerica, MA, USA) as previously reported [25]. Four different fields were measured for each sample, in three independent experiments with about 80–100 total cells.

### 2.7. RUNX2 Transfection

RUNX2 transfection was performed using Lenti ORF particles, RUNX2 (Origene Technologies, Rockville, MD, USA), according to the manufacturer’s instruction. Briefly, when del-RUNT cells were at 60% confluence, they were incubated with and without lentiviral particles and polybrene (1000× stock solution (8 mg/mL). After 4 h, the medium was replaced, and protein detection was performed after 3 days. For each condition, three independent experiments were performed.

### 2.8. Total RNA Extraction and Reverse Transcription (RT)

Total RNA extraction and RT were performed as previously reported [11].

### 2.9. Real Time RT-PCR

PCRs were performed in a total volume of 25 µL containing PCR Master with ROX premixed with SYBR Green and 20 ng of cDNA from each sample. The following primer sets were used: *STMN1* (FW: AGATGTACTTCTGGACTCAC–RV: GATCAGACCAGGTAATCAATG)*–SSBP1* (FW: GGTGATGTCAGTCAAAAGAC–RV: TCACATATTGATATGCCACG)–*SOX9* (FW: CGGAGGAAGTCGGTGAAG–RV: CTGGGATTGCCCCGAGTGCT). For *p53* expression analysis, we used the HsTP53-1-SG Quanti Tect Primer Assay (Qiagen, Venlo, Netherlands). Real Time RT-PCR reactions were performed as previously reported [11]. Gene expression was normalized to housekeeping genes (Beta-2 Microglobulin (*B_2_M*) and Glyceraldehyde-3-Phosphate Dehydrogenase (*GAPDH*)) using the relative fold expression differences. Ct values for each reaction were determined using the TaqMan SDS analysis software. For each amount of RNA, three independent experiments with three replicates for each sample were performed.

### 2.10. Migration Assay

A scratch test was used to evaluate cell migration capability. Cells were cultured in 48-well plates and grown to 80% confluence. The scratch track was produced by using a 200-µL pipette tip. The supernatants were removed, and the plates were washed with PBS and grown in fresh medium. Cell migration was observed in time-lapse (EVOS), and the relative migration was calculated as previously reported (8), i.e., as relative migration distance (RMD) (%) = 100 (A − B)/A, with A and B representing the width of cell scratches before and after incubation, respectively. Data were obtained from three independent experiments.

### 2.11. Western Blot

Protein concentrations were determined with the BCA assay (Thermo Scientific, Waltham, MA, USA). Each lysate was separated by sodium dodecyl sulfate–polyacrylamide gel electrophoresis (SDS-PAGE) and probed with rabbit monoclonal antibodies against RUNX2 (Cell Signaling, 8486), mouse monoclonal antibodies against RUNX2 (D130-3, clone 8G5; MBL), N-Cadherin (EPR1791-4), anti E-Cadherin antibody (EP700-Y Abcam, Cambridge, UK), anti-Vimentin antibody (EPR3776 Abcam) anti p53 (pAb122 Abcam), and GAPDH SC-166545 (Santa Cruz Biotechnology, Dallas, TX, USA) according to manufacturer’s instructions. Immuno-reactive proteins were detected using an enhanced chemiluminescence reagent (ECL, Millipore, Burlington, MA, USA) according to the manufacturer’s instructions. Images were captured either on film or by LAS4000 Digital Image Scanning System (GE Healthcare, Little Chalfont, UK). Densitometric analysis was performed by using ImageJ software (v 1.43o8, NIH, Bethesda, MD, USA), and the relative protein band intensity was normalized to GAPDH and expressed as OD ratio. Data were obtained from three independent experiments.

### 2.12. Zebrafish Handling and Xenotransplantation

Zebrafish experiments were performed at the Zebrafish Centre of the University of Padova, Italy, under ethical authorization 407/2015-PR.

Zebrafish embryos were obtained from natural spawning of nacre adults (ZFIN database ID: ZDB-ALT-990423-22), raised according to standard protocols [26], and staged according to Kimmel et al. [27].

For xenotransplantation, embryos were mechanically dechorionated at 2 dpf (days post-fertilization), anesthetized with tricaine 0.16 mg/mL, and placed along the lanes of a microinjection mold, immersed in 2% methylcellulose/fish-water. Melanoma cells were counted, suspended at a density of 1 × 10^6^/mL, and stained with 5 μg/mL of Vybrant Cell-Labeling Solution (Thermo Fisher Scientific, Milan, Italy) either in red (DiI stain) or green (DiO stain) for 20 min at 37 °C, according to the manufacturer’s protocol.

Stained cells were loaded into a glass capillary needle and microinjected into the yolk (about 50 cells/embryo), using a WPI PicoPump apparatus. Xenotransplanted embryos (A375 transplanted zebrafish number: 74; del RUNT transplanted zebrafish number: 48) were grown at 33 °C, monitored daily, and documented from the injection day up to 1 week post-injection (experimental endpoint, 9 dpf).

Imaging was performed using a Leica MZFLIII fluorescence dissecting microscope equipped with a Leica DFC7000T camera (Leica Microsystem, Milan, Italy). Panels were assembled using Adobe Photoshop CC v. 14.0 (64 bit) (Adobe Inc., San Jose, CA, USA).

### 2.13. Statistical Analysis

Results were expressed as mean ± S.E. Paired *t*-test was used to compare the variation of the same variable between two groups. For multiple comparisons, statistical analysis was assessed by one-way analysis of variance (ANOVA). Differences between groups yielding a statistical significance with *p* < 0.05 were tested with Bonferroni as a post hoc test. Analyses were applied to experiments carried out at least three times. Statistical analyses were performed using SPSS for Windows, version 16.0 (SPSS Inc., Chicago, IL, USA).

This section may be divided by subheadings. It should provide a concise and precise description of the experimental results, their interpretation, as well as the experimental conclusions that can be drawn from them.

## 3. Results

### 3.1. Bioinformatic Analysis of RUNX2 using the Cancer Genome Atlas (TCGA) Skin Cutaneous Melanoma Dataset

**Figure 1:**
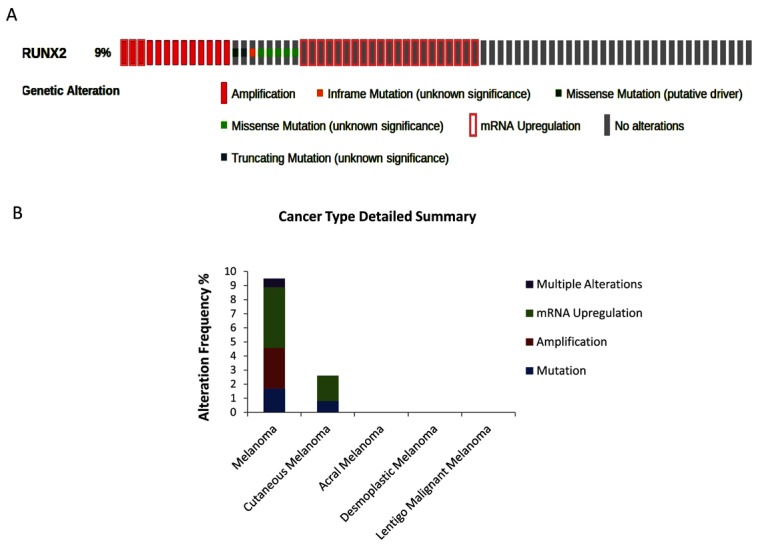
Genomic alterations in TCGA samples of patients affected by Skin Cutaneous Melanoma (SCKM). OncoPrint: graphical summary of genomic alterations in *RUNX2* detected in 42 of 478 samples. A: the figure shows the samples with alterations (**A**); Distribution of alteration frequency in *RUNX2*: mRNA upregulation: 4.39%, gene amplification: 2.09%, mutation: 1.67%, and, finally, multiple alterations (0.63%) (**B**).

The bioinformatic analysis by cBioPortal showed that *RUNX2* is altered in 9% of TCGA samples (42/478) (Figure 1A).

**Figure 2:**
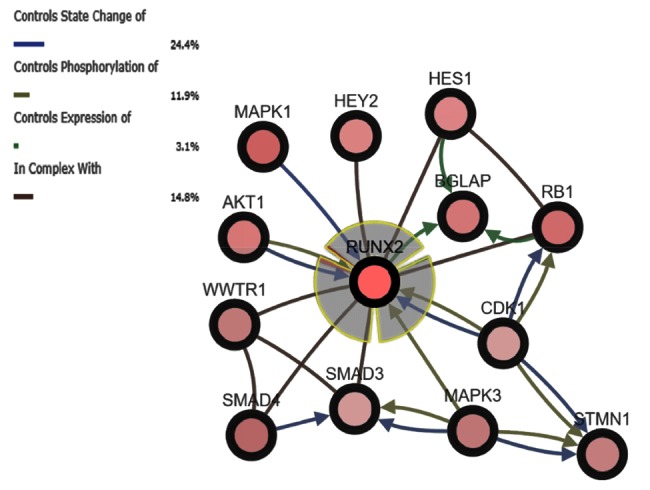
Network Analysis by cBioportal and genomic alterations spectra. The Network depicts RUNX2 and the most relevant interacting genes with the highest genomic alteration in SKCM. The alteration spectrum is reported for each gene. The state of change of RUNX2 is controlled via phosphorylation by *AKT1*, *CDK1*, and *MAPK3* products. RUNX2 can form different types of complex with product of HES1, HEY2, RB1, SMAD3, SMAD4, and WWTR1. Concerning transcriptional modulation, RUNX2 controls *BGLAP* gene expression. Finally, *STMN1*, *BGLAP*, *HEY2*, *MAPK1*, *SMAD4*, and *WWTR1* are neighbor genes.

**Figure 3:**
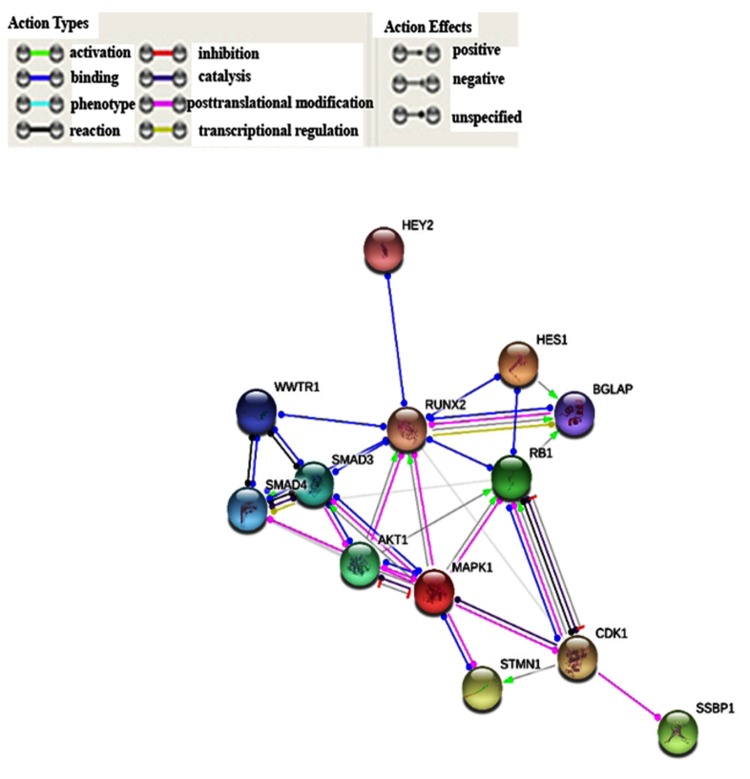
Functional proteins interaction classified according to activity and effect. PPI enrichment *p*-value: 1.03 × 10^−7^.

mRNA upregulation (4.39%) and gene amplification (2.09%) were the prevailing alterations, followed by mutation (i.e., missense, truncating, in-frame, and all other types of mutations) (1.67%), and, finally, multiple alterations (0.63%) (Figure 1B). In addition, the analysis showed that *RUNX2* interacts with the following 12 partner genes products (Figure 2):

In particular: *MAPK1* (Mitogen-activated protein kinase 1; 14% altered), *RB1* (Retinoblastoma 1; 12% altered), *SMAD4* (SMAD family member 4; 12% altered), *AKT1* (AKT Serine/Threonine Kinase 1; 11% altered), *WWTR1* (WW domain containing transcription regulator 1; 9% altered), *STMN1* (stathmin 1, 6.5% altered), *BGLAP* (Bone Gamma-Carboxyglutamate Protein; 8% altered), *CDK1* (Cyclin-dependent kinase 1; 7% altered), *MAPK3* (Mitogen-activated protein kinase 3; 7% altered), *SMAD3* (SMAD family member 3; 7% altered), *HEY2* (Hes Related Family BHLH Transcription Factor With YRPW Motif 2; 6% altered), and *HES1* (Hes Family BHLH Transcription Factor 1; 6% altered). It is noteworthy that RUNX2 showed a statistically significant co-occurrence of alterations with the genes *WWTR1* (log 242 OR = 1.466; p(logOR = 1.466; *p* < 0.001) and *SMAD3* (logOR = 1.108; *p* < 0.05). Finally, the STRING analysis summarized the herein-reported genes into the following functional gene ontology terms: regulation of transcription (GO.0045944), signal transduction (GO.0009966), cell proliferation (GO.0008284), and cell differentiation (GO.0045597). Moreover, the PPI (Protein Protein Interaction) enrichment analysis confirmed the strong association among them (*p*-value = 3.65 × 10^−8^) (Figure 3 and Figure S1).

### 3.2. Generation of del-RUNT Cells

**Figure 4:**
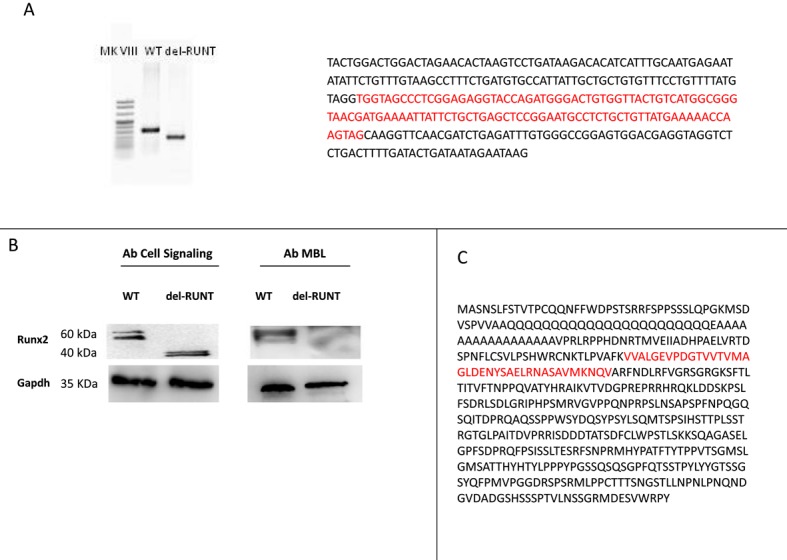
A clone with an in-frame 114 bp deletion in the RUNT domain was obtained by CRISPR/Cas9. On the left, agarose gel showing PCR products (WT: 340 and del-Runt 226 Bp). On the right, the Sanger sequence of the PCR products with the 114 bp deletion highlighted in red. (**A**) Western Blot (WB) of RUNX2 in WT and del-RUNT cells. A shorter protein lacking 38 residues was observed by using the antibody produced by Cell Signaling manufactures; No Runx2 protein was detected in del-Runt cells by using the MBL antibody in WB (**B**); On the right, the Runx2 protein sequence (UniProtKB/Swiss-Prot: Q13950.2); the 38 aa deletion is highlighted in red in the protein context (**C**).

We used the CRISPR/Cas9 technique to knockout the RUNT domain, which is the invariant and highly conserved DNA binding domain of RUNX2. After selection and expansion of clones (*n* = 11), we performed a PCR analysis followed by Sanger sequencing of the specific *RUNX2* genomic region in all clones. We identified and isolated a single clone with the desired 114 bp in frame deletion in the RUNT domain (Figure 4A), which we called “del-RUNT”.

The in-frame deletion, corresponding to 38 amino acids, leads to the synthesis of a shorter protein, detected by the Cell Signaling Runx2 antibody B and Figure S2) in which the RUNT region was missing in the middle (Figure 4C). Conversely, using the MBL RUNX2 antibody, previously used by Meyer et al. [28], against the region where the 38 amino acids deletion occurred, no protein was detected, as expected (Figure 4B); this further confirmed the expression of a deleted form of RUNX2.

### 3.3. The RUNT Domain Increases Cell Viability and Proliferation and Reduces Apoptosis

**Figure 5:**
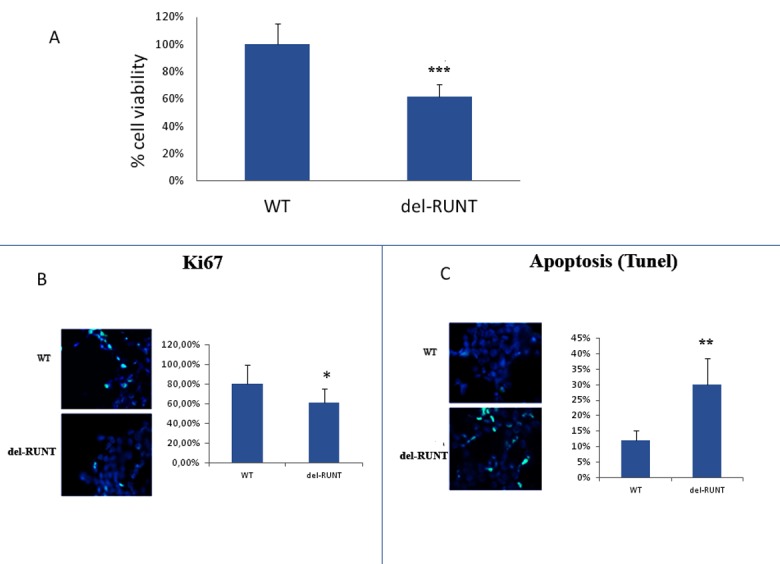
Significantly lower cell viability evaluated by the XTT test (**A**, *** *p* < 0.0001) and proliferation, evaluated in immunofluorescence by ki67 positive cells (**B**, magnification X40, * *p* < 0.05), was observed in del-RUNT compared to wild-type cells. On the contrary, apoptosis, evaluated by the TUNEL test, was significantly higher in del-RUNT cells (**C**, magnification ×40, ** *p* < 0.01). The graphs for ki67 positive cells and apoptosis were generated by three independent experiments using 80–100 total cells per experiment.

To evaluate the biological effects of the deletion in the RUNT domain, we analyzed cell viability using the XTT test. After 48 h, we observed a viability reduction of about 40% in del-RUNT cells compared to WT A375 parental cells (*p* < 0.0001) (Figure 5A).

In order to assess if the reduced viability was the consequence of a reduced proliferation or an increased level of apoptosis, we performed the Ki67 expression and TUNEL analyses. As showed in Figure 5B, we observed a significantly reduced percentage of nuclei expressing Ki67 (61.2%; SD ± 12%) in del-RUNT cells compared to WT (80.4%, SD ± 15%) (*p* < 0.05). In addition, the percentage of positive apoptotic nuclei was higher in del-RUNT cells (30%; SD ± 8%) compared to WT (12%; SD ± 10) (*p* < 0.01) Figure 5C.

**Figure 6:**
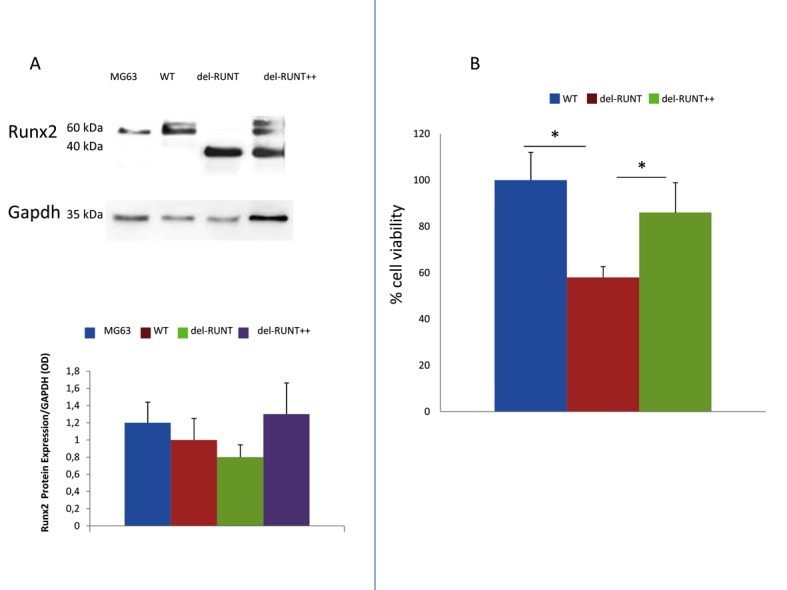
Western blot analysis of del-RUNT clones. MG63: osteosarcoma cell line (used as positive control for Runx2 protein); WT: A375 cell line; del-RUNT, clone with inframe deletion of RUNT; del-RUNT++: same clone, reexpressing wild-type RUNX2 after transduction with Lenti ORF viral vector. GAPDH, internal control used to normalize protein expression. The re-expression of the wild protein after transduction with Lenti ORF viral vector particles expressing RUNX2 (**A**) induced an increased cell viability compared to del-RUNT cells (**B**). (Data were obtained by three independent experiments * *p* < 0.0001).

The normal protein was re-expressed in cells transfected with RUNX2- Lenti ORF particles (del-RUNT++) Figure 6A and Figure S2). Interestingly, del-RUNT++ cells showed an increased viability compared to del-RUNT cells (Figure 6B).

### 3.4. RUNT Modulation of Genes Involved in Metastatic Progression

**Figure 7:**
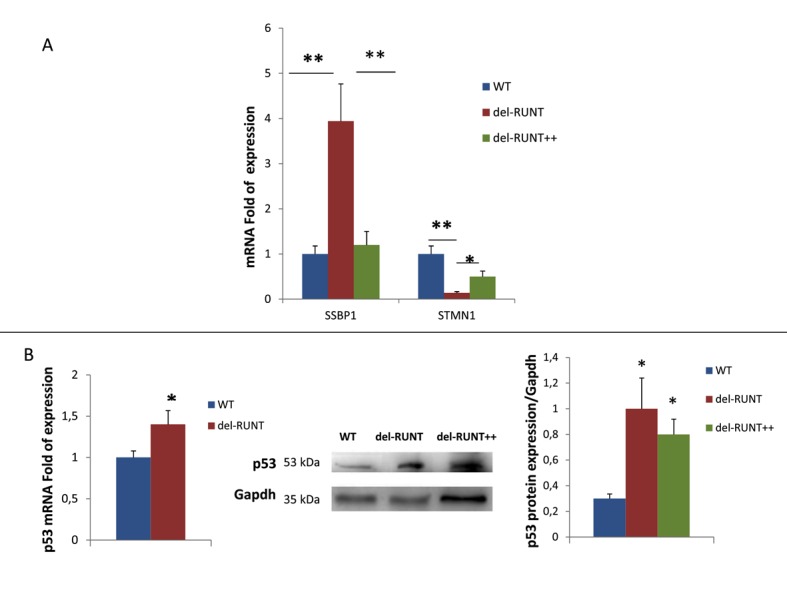
The mRNA expression of *SSBP1*, a gene encoding for a protein involved in p53 stabilization, evaluated by Real Time PCR and normalized to the housekeeping genes *B2M* and *GAPDH*, was significantly higher in del-RUNT compared to WT cells. Conversely, the mRNA expression of *STMN1*, involved in melanoma transformation, was reduced in del-RUNT cells. Expression of *SSBP1* and *STMN1* was restored in del-RUNT++ melanoma cells (**A**). *P53* gene expression was increased in del-RUNT compared to WT cells (**B**). In addition, the WB analysis showed an increased expression of the p53 protein in del-RUNT cells and in del-RUNT++ cells compared to WT (A375 cells) (**B**) * *p* < 0.05; ** *p* < 0.001.

Among the partner genes of RUNX2 identified by bioinformatic analysis, we chose to investigate the expression of the *SSBP1* gene, involved in p53 transcriptional activity, and of the *STMN1* gene, considered a melanoma oncogene, in order to evaluate the effects of RUNT deletion on malignant transformation. We observed a higher expression of *SSBP1* in del-RUNT (3.94 fold of expression; SD ± 1.41; *p* < 0.001) compared to the expression in WT cells (SD ± 0.99) (*p* < 0.01) (Figure 7). On the contrary, the expression levels of *STMN1* were lower in del-RUNT (0.14 fold of expression; SD ± 0.14) compared to the expression levels in WT (SD ± 0.29; *p* < 0.001) (Figure 7).

RUNX2 transfection restored the expression levels of target genes in del RUNT cells. Thus, when we performed gene expression analysis by Real Time PCR in del-RUNT++ cells, we observed a lower expression of *SSBP1* (1.2 fold of expression; SD ± 0.62; *p* < 0.001) and a higher expression of *STMN1* (0.55 fold of expression; SD ± 0.2; *p* < 0.05) compared to del-RUNT cells (Figure 7A). Interestingly, *p53* expression, evaluated by Real Time PCR, was higher in del-RUNT compared to WT cells. Consequently, p53 protein levels, evaluated by Western Blotting, were higher in del-RUNT and in del RUNT++ cells, confirming the positive modulatory effect of *SSBP1* on p53 expression (Figure 7B).

### 3.5. RUNT Domain Promotes Epithelial Mesenchymal Transition and Migration Ability

**Figure 8:**
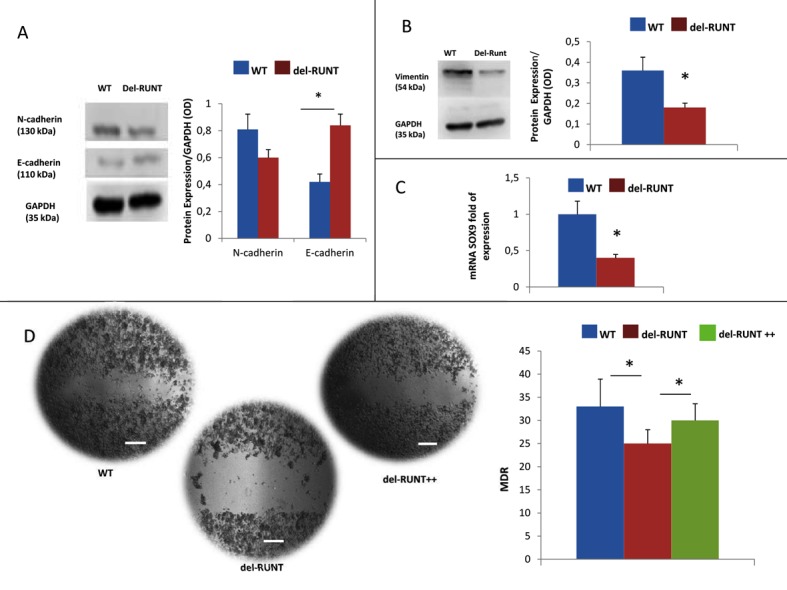
Western blot analysis comparing N and E cadherin expression in WT (A375) and del-RUNT cells. GAPDH, internal control. The deletion of the RUNT domain in RUNX2 affected the EMT, as shown by the higher expression of E cadherin and the lower expression of N cadherin normalized to GAPDH and expressed as OD (Optical density) (**A**). The 38 aa deletion is highlighted in red. Vimentin expression was also reduced in del-RUNT cells as shown in western blot analysis and normalized to GAPDH control (**B**). mRNA expression analysis, evaluated by Real Time PCR and normalized to the housekeeping genes *B2M* and *GAPDH*, showed the downregulation of *SOX9*, a gene involved in the EMT process, as well (**C**). Migration ability (expressed as relative migration distance (RMD)) was also reduced in del-RUNT cells (**D**). The re-expression of RUNX2 in del-RUNT cells (del-RUNT ++) restored cell migration capability. * *p* < 0.05; scale bar 100 µm.

We analyzed vimentin, N, and E cadherin expression by Western Blotting, and *SOX9* gene modulation by Real Time PCR in WT and del-RUNT cells, to evaluate the EMT phenotype in both cell lines. In del-RUNT cells, we observed a higher E cadherin expression (0.89 OD ratio; SD ± 0.2) compared to WT (0.48 OD ratio; SD ± 0.18), and this increase was statistically significant (*p* < 0.05) (Figure 8A).

On the contrary, del-RUNT cells expressed lower levels of N cadherin (0.68 OD ratio; SD ± 0.15) compared to WT (0.87 OD ratio; SD ± 0.14), even if the difference was not statistically significant (Figure 8A). Interestingly, vimentin expression was lower in del-RUNT cells (0.21 OD ratio; SD ± 0.06) compared to WT cells (0.35 OD ratio; SD ± 0.08), (*p* < 0.05) (Figure 8B). In addition, the mRNA expression of *SOX9* gene, involved in the EMT process, was decreased in del-RUNT cells (0.42 fold of expression; SD ± 0.12) compared to WT cells (1 fold of expression; SD ± 0.02) (*p* < 0.001) (Figure 8C).

In order to evaluate cells migratory capability, we performed the scratch test and we analyzed the relative migration distance (RMD) after 48 h of cell growth. We observed a lower migratory capacity for del-RUNT cells (21.8%; SD ± 7%) compared to WT cells (33.4%; SD ± 10%) (Figure 8D). The re-expression of RUNX2 in del-RUNT cells (del-RUNT++ cells) restored cells’ migratory capability in vitro.

The reduced migration ability observed in vitro was also detected in vivo. As zebrafish is considered a useful in vivo model to investigate the progression of melanoma [29,30], we exploited this system to evaluate the ability of del-RUNT cells to form metastases when xeno-transplanted in zebrafish. Wild-type melanoma cells (A375, stained in red) and del-RUNT cells (3G8, stained in green) were injected in the yolk of zebrafish embryos (transplanted zebrafish number: 22; A375 transplanted zebrafish number: 74; del RUNT transplanted Zebrafish number: 48), and metastasis formation was monitored between the 5th and 7th day after injection.

**Figure 9:**
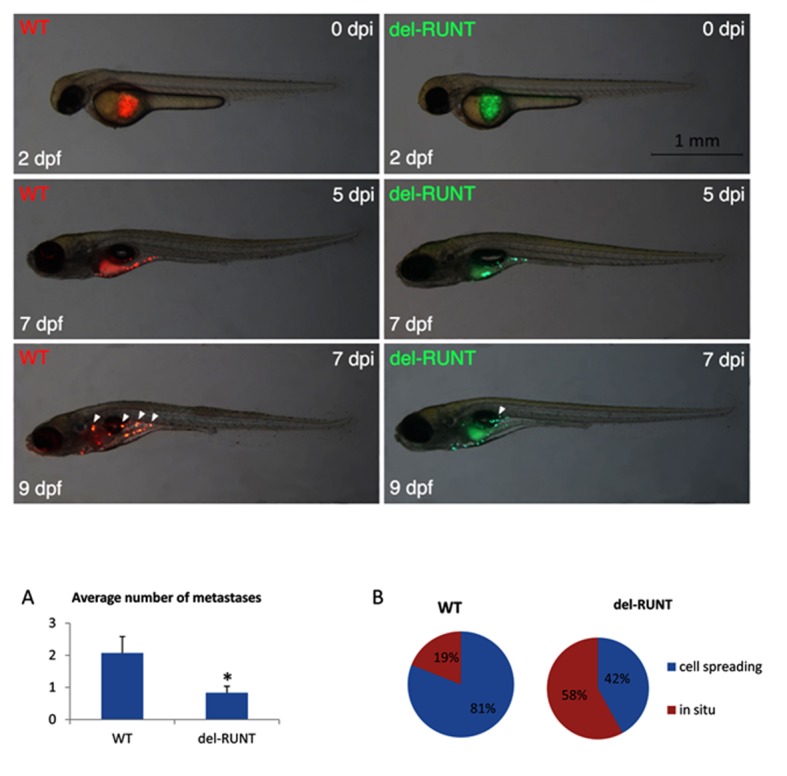
Differential migratory ability of wild-type and del-RUNT cells in zebrafish xeno-transplants. Representative images show wild-type (WT, stained in red) and del-RUNT (del-RUNT, stained in green) cells xeno-transplanted in zebrafish embryos 2 days post fertilization (dpf). At day 0 post injection (dpi), transplanted cells are still located in the yolk. At 5 dpi, cells appear more dispersed in the yolk of 7 dpf larvae. At 7 dpi/9 dpf, initial metastases (indicated by white arrowheads) are detectable in WT and, to a lesser extent, in del-RUNT- transplanted larvae. All views are lateral, anterior to the left. Charts on average number of metastases and cell spreading, calculated at 9 dpf in WT (N:22) and del-Runt (N:12) transplanted zebrafish, are shown in (**A**) and (**B**), respectively. * *p* < 0.05.

At the experimental endpoint (9th day after injection), the number of zebrafishes (controls and transplanted) was reduced exclusively because of mortality (around 60% for all groups). Therefore, at the 9th day after injection, the average number of metastases was 2.07 for zebrafishes transplanted with A375 wild-type melanoma cells, while a number of 0.83 metastases were observed for zebrafishes transplanted with del-RUNT cells, indicating RUNT domain KO reduces the probability of cell spreading (Figure 9A). Interestingly, A375 wild-type melanoma cells migrated in 81% and remained in situ in 19% of transplanted zebrafishes, while del-RUNT cells migrated in 58% and were still in situ in 42% of transplanted zebrafishes, confirming the reduced migratory ability of melanoma cells lacking the RUNT domain (Figure 9B).

## 4. Discussion

The bioinformatic interrogation of the SKCM TCGA dataset highlights a strong correlation between the query genes and melanoma (false discovery rate (fdr): 0.000534) and showed that *RUNX2* was altered in 9% of subjects affected by melanoma. Overexpression, due to mRNA upregulation and gene amplification, is the most frequent alteration indicating its oncogenic role, almost certainly in the late stages of tumor promotion and progression, EMT, and metastasis. In *SCKM*, *RUNX2* was part of a network including other interacting altered genes (*HES1*, *HEY1*, *BGLAP*, *SMAD3*, *SMAD3*, *WWTR1*, *MAPK1*, *MAPK3*, *AKT1*, *CDK1*, *SSBP1*, *STMN1*, and *RB1*), partaking in regulation of transcription, signal transduction, cell proliferation, vascular endothelial growth factor receptor signaling, and differentiation pathways (such as NOTCH-mediated HES/HEY, TGF-β/activin, MAPK, WNT, and PI3K/AKT pathways). The cBioPortal estimates the likelihood, by determining the Odds Ratio (OR), that alterations in paired queried genes occur in a mutually exclusive fashion or in co-occurrence. Mutual exclusion analysis statistical significance is evaluated by Fisher’s exact test and the Bonferroni correction. Interestingly, RUNX2 alterations are concomitant with *SMAD3* (logOR = 1.108; *p* < 0.05; alteration percentage 7%) and *WWTR1* (log OR = 1.466; *p* < 0.001; 9%). WWTR1 interacts with RUNX2 to promote gene expression (pathway id: R-HSA-2032785) and encodes a transcriptional co-activator playing a crucial role in the Hippo, TGF-beta, and canonical Wnt pathways. Furthermore, WWTR1 promotes EMT as well [31]. It may be speculated that the potentiating effects of *RUNX2* and *WWTR1* on melanoma are stronger when they are concomitantly altered. Another important issue is the role of RUNT, the DNA binding domain of *RUNX2*. Up to now, RUNT has not been investigated, and data on its involvement in malignant transformation are lacking. In this study, we focused on how RUNX2 acts by deleting its RUNT domain using CRISPR/Cas9 and then analyzing possible changes in melanoma cells. Notably, the CRISPR/Cas9 approach allows one to scan specific gene domains. This aspect is particularly advantageous when investigating the action of transcription factors.

*RUNT* deletion altered the cellular phenotype. In fact, viability was reduced in del-RUNT cells, and we observed a higher apoptotic rate in these cells compared to WT cells. This suggests that the RUNT domain may be involved in cell protection from apoptosis. Shin and co-workers used an integrative genome-wide approach combining ChIP-seq and RNA-seq to identify downstream targets of RUNX2 involved in anti-apoptotic activity [32]. Interestingly, these authors identified that *MYC* is a direct target of RUNX2, and they also demonstrated that MYC is required for the survival of osteosarcoma cells [32]. In addition, RUNX2 is a negative regulator for p53 in response to DNA damage [33]; its inibitory effect is mediated by the interaction with HDAC6 [33]. In addition, we observed the upregulation of *SSBP1* gene expression in del-RUNT cells. The SSBP1 regulates both the stability and the transcriptional activity of *p53* [4], and, in fact, p53 expression was higher in del-RUNT cells compared to WT. Then, the observed upregulation of *SSBP1* and *p53* in del-RUNT cells corroborates the idea that RUNX2 plays an anti-apoptotic role in melanoma.

Melanoma cells’ malignancy may be ascribed to their high ability to acquire a metastatic phenotype. The incidence of metastatic melanoma has increased in the last decades; it causes most skin cancer deaths. Therefore, the possibility to prevent or reduce the cellular migratory ability represents an important aim to counteract melanoma. In del-RUNT cells, we found reduced expression of RMD and downregulation of *STMN1*, a gene involved in cell migration. The re-expression of normal RUNX2 restored migratory ability. On the other hand, it has been shown that miR-203 inhibits the migration and invasion of osteosarcoma cells by targeting *RUNX2* mRNA [34]. In prostate cancer, miR-466, by directly repressing the osteogenic transcription factor *RUNX2*, inhibits tumor growth and metastases [35], highlighting the role of RUNX2 in inducing the malignant phenotype.

It has been shown recently that the knockdown of *RUNX2* in esophageal carcinoma cells significantly inhibits cell migration and invasion, suppresses tumor formation in vivo, and increases apoptosis [36]. Here, we show that the mere deletion of 114 bp in the RUNT domain was able to induce all the observed effects such as the reduction of proliferation and cell migration capability, as well as the increase of apoptosis. The EMT process was also affected by the deletion in the RUNT domain. In a previous work, Meyer et al. used the CRISPR/Cas9 system to knockout the *RUNX2* gene in osteoblasts cells [28]. The authors introduced a 33-bp in-frame deletion in exon 3, and the antibody they used did not recognize the edited RUNX2 protein. Similarly, we did not detect the edited protein in our model when we used the same antibody used by Meyer. On the contrary, by using an antibody against a distal region of the deletion, we identified a shortened form of RUNX2 protein. The fact that two different groups, our and Meyer’s, were able to obtain only in-frame deletion mutants makes us wonder whether the complete knockout of *RUNX2* might be lethal, impacting cell viability.

The reduced ability to migrate was confirmed in an in vivo model: the number of A375 wild-type cells able to migrate in zebrafish was higher compared to cells harboring the deletion.

Furthermore, the functionality of the protein with the partially removed RUNT domain was compromised as shown by the observed data, and the malignant features of melanoma cells were restored with the re-expression of WT RUNX2. In addition, this is the first study showing the effects of this binding domain on genes involved in cancer, such as *STMN1* or *SSBP1*, which are not direct targets of RUNX2. All these findings suggest a pivotal role of the RUNT domain in maintaining and, probably, inducing the transformed phenotype of melanoma cells, by altering different pathways.

In conclusion, our study suggests that RUNT domain may be an ideal oncotarget in melanoma cancer cells as well as in a variety of other common cancers, in which RUNX2 has been found abnormally expressed.

## Supplementary Materials

The following are available online at http://www.mdpi.com/2073-4409/7/11/220/s1, Figure S1: Protein-protein functional association, Figure S2: Representative image of a Western blot.
