# Supplementary material for: New Insights into the Runt Domain of RUNX2 in Melanoma Cell Proliferation and Migration
Source: Cells. 2018 Nov 20;7(11):220. doi: 10.3390/cells7110220 (PMC6262450; doi:10.3390/cells7110220)
Supplement: Supplementary file 1 [file cells-07-00220-s001.pdf]

New Insights into the Runt Domain of RUNX2 in Melanoma Cell Proliferation and Migration

Michela Deiana [1,2], Luca Dalle Carbonare [2], Michela Serena [1], Samuele Cheri [1,2], Francesca Parolini [1], Alberto Gandini. [3], Giulia Marchetto [2], Giulio Innamorati [3], Marcello Manfredi [4], Emilio Marengo [4], Jessica Brandi [5], Daniela Cecconi [5], Antonio Mori [1], Maria Mihaela Mina [2], Franco Antoniazzi [3], Monica Mottes [1], Natascia Tiso [6], Giovanni Malerba [1], Donato Zipeto [1] and Maria Teresa Valenti [2,*]

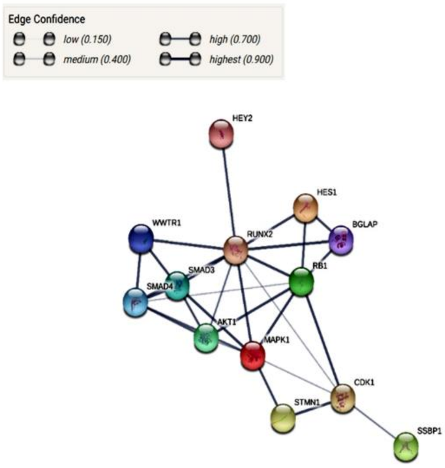

Figure S1. Protein-protein functional association based on confidence. Line thickness indicates the strength of data support. PPI enrichment p-value: 1.03e-07.

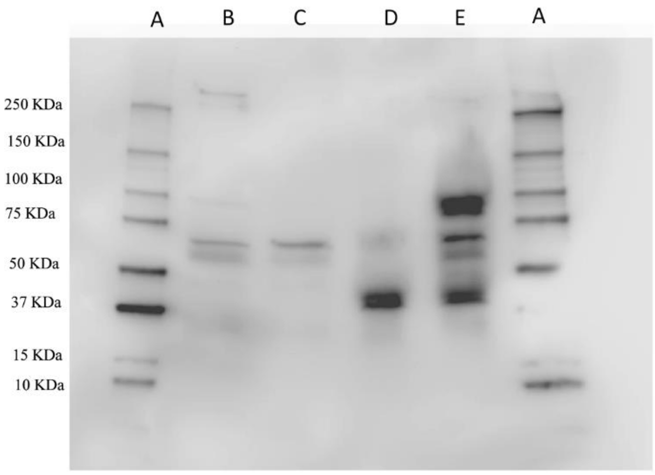

Figure S2. Representative image of a Western blot reaction against Runx2. Lanes A: MW; Lane B: MG63 osteosarcoma cell line; Lane C; WT: A375 cell line; Lane D: del-RUNT, clone with in-frame deletion of RUNT; Lane E: del-RUNT++: same clone, re-expressing wild-type RUNX2 after transduction with Lenti ORF viral vector (note around 75 kDa the RUNX2 mGFP-tagged).
